# Cross-cultural adaptation of an environmental health measurement instrument: Brazilian version of the health-care waste management • rapid assessment tool

**DOI:** 10.1186/s12889-016-3618-4

**Published:** 2016-09-05

**Authors:** Eliana Napoleão Cozendey-Silva, Cintia Ribeiro da Silva, Ariane Leites Larentis, Julio Cesar Wasserman, Brani Rozemberg, Liliane Reis Teixeira

**Affiliations:** 1 Sergio Arouca National School of Public Health, Oswaldo Cruz Foundation, ENSP/FIOCRUZ, Rua Leopoldo Bulhões 1480, Prédio Primeiro de Maio. Manguinhos, CEP 21041-210. Rio de Janeiro, RJ Brazil; 2 Municipal Health Foundation of Niteroi, VIPAHE/FMS, Av Ernani do Amaral Peixoto, 171, Centro–CEP 24020-071. Niterói, RJ Brazil; 3 Network of Environment and Sustainable Development (REMADS-UFF), University Federal Fluminense, Av. Gal. Milton Tavares de Souza, s/n. Praia Vermelha, CEP 24210-346. Niterói, RJ Brazil

**Keywords:** Cross-cultural comparison, Rapid evaluation, Waste management, Medical waste, Environmental health, Health care evaluation, Public policies

## Abstract

**Background:**

Periodic assessment is one of the recommendations for improving health-care waste management worldwide. This study aimed at translating and adapting the Health-Care Waste Management - Rapid Assessment Tool (HCWM-RAT), proposed by the World Health Organization, to a Brazilian Portuguese version, and resolving its cultural and legal issues. The work focused on the evaluation of the concepts, items and semantic equivalence between the original tool and the Brazilian Portuguese version.

**Methods:**

A cross-cultural adaptation methodology was used, including: initial translation to Brazilian Portuguese; back translation to English; syntheses of these translation versions; formation of an expert committee to achieve consensus about the preliminary version; and evaluation of the target audience’s comprehension.

**Results:**

Both the translated and the original versions’ concepts, items and semantic equivalence are presented. The constructs in the original instrument were considered relevant and applicable to the Brazilian context. The Brazilian version of the tool has the potential to generate indicators, develop official database, feedback and subsidize political decisions at many geographical and organizational levels strengthening the Monitoring and evaluation (M&E) mechanism. Moreover, the cross-cultural translation expands the usefulness of the instrument to Portuguese-speaking countries in developing regions.

**Conclusion:**

The translated and original versions presented concept, item and semantic equivalence and can be applied to Brazil

## Background

The promotion of environmental health and the proper management of waste, especially the most dangerous types, are worldwide issues. Environmental contamination and occupational accidents caused by improper health-care waste management (HCWM) are on-going challenges [1–3], especially in less developed countries [4, 5]. To address these concerns, a number of political and legal mechanisms have been developed, to protect both the environment and human health against the risks associated with health-care waste (HCW) [4–7].

The hazardous nature of HCW is associated not only with blood-borne infections, mainly caused by sharps injuries, but also with diseases related to the contamination of soil, water and air caused by the inadequate treatment and/or final disposal of HCW [1–3, 8–12]. In less developed countries these problems are recurrent [4, 13] due to the poor access and precarious situation of health basic services, urban infrastructure, water supply, sewage system and waste collection, among others. In relation to HCWM, many health-care facilities (HCF) wrongly dispose their hazardous waste with the ones similar to household waste, others burn them in open dumps or in incinerators without the necessary devices for environmental contamination control, exposing the nearby communities to their toxic emissions (dioxins, mercury and particulate matter, for example) [4].

Aware of the local problems and global dimensions in the last few decades, both national and international organizations have been intensifying the effort to improve management practices [4, 14] aimed at global disease burden reduction. Health-care organizations have committed to finding solutions to the challenge of continually improving their environmental performance and to achieving measurable results [15].

Regulatory organizations, such as the Brazilian Health Surveillance Agency (Anvisa) [16], the National Environmental Council (Conama) [17] and the National Nuclear Energy Commission (Cnen) [18], are based on a mediator and regulator model, and are responsible for national policies concerning HCWM, from its inception to its final disposal (“cradle to grave” or “cradle to cradle” for recyclable waste).

These organizations have political, financial, regulation and management autonomy and through two main Federal Acts (Anvisa 2004 and Conama, 2005) [16, 17] determine that HCW generators are responsible for their correct waste management and must elaborate a free public access document relating to HCW management including the monitored indicators. The technological information center (databases) [19] of the Unified Health System – SUS (Public health care system for Brazilians and foreigners travelers over the country) [20] has an available field [21] where HCF managers must inform the category and amount of HCW generated by them.

The National Solid Waste Policy [22] is guided by the principles of sustainability and environmental protection. Although it does not deal specifically with healthcare waste, it increases the responsibility of every waste generator to provide proper final disposal. According to this policy, hazardous waste generators must develop specific waste management plans, taking the wastes’ inherent risks into consideration [23].

In this context, the main Brazilian legal regulations for HCWM [16, 17, 24] establish guidelines regarding the elaboration, and request the implementation and development of a Health-care Waste Management Plan (HCWMP) in every HCF. The HCWMP is part of an integrated management system for environmental health, and must include aspects related to all stages of waste management, professional awareness and qualifications, and occupational health and safety, as well as monitoring and evaluation (M&E) methods for proper health-care waste management [16, 24].

As a consequence of the HCWMP, researchers, interlocutors and managers have demonstrated an increasing interest in developing an integrated system for HCWM assessment [15]. Nonetheless, to our knowledge, in Brazil, no assessment tools for the straightforward evaluation of an integrated HCWM plan, adapted to various data collection modes and scopes, have yet been developed.

Among the international proposals identified, the Health-Care Waste Management – Rapid Assessment Tool (HCWM-RAT) [25] is considered the best for satisfying the evaluation criteria: scope, field investigation, methodology, robustness, origin and adaptability to the Brazilian context. This tool is part of an overall strategy developed by WHO to achieve a reduction in the disease burden attributed to inadequate health-care waste management. Assessment-in-context reviews are recommended by the WHO as a requirement for improving HCWM systems, since their implementation is unsatisfactory in health care institutions in many countries throughout the world [4, 25].

The (HCWM-RAT) [25] structure is based on a Rapid Evaluation Method (REM) and methodological triangulation: that is, a combination of qualitative and quantitative methods for collecting and analyzing data [26, 27]. This structure makes the HCWM-RAT distinct from other commonly used standard tools for gathering information on HCWM, which are based only on a check-list survey, and lack the perspective of a participative and emancipatory assessment [28].

Research in the field of environmental health evaluation, particularly for HCWM, is critical, because of the need for improving control of the spread of pollutants [29]. Cross-cultural adaptation of measuring instruments for HCWM, however, is scarce, and this study found none regarding Brazilian instruments. Furthermore, Brazil has been receiving thousands of Colombian refugees [30, 31], as well as immigrant workers from Africa and Haiti, and tourists, due to recent sports events. Brazilian health facilities must therefore be prepared not only for foreign visitors’ health assistance but also for the safe management of any HCW that might be produced.

Brazilian political structure is aligned with the principles of the World Health Organization (WHO) [4] to achieve safe and sustainable HCWM. Moreover, underlines the importance of periodic assessment to generate reliable information that will support interventions focused on ensuring best practices and waste reduction. However, through a large consensus from the literature, the use of such measuring instrument (questionnaire) made in other nations should be preceded by cross-cultural investigation (cross-cultural adaptation process) [32–37].

So, as this tool was originally developed in the U.S., cross-cultural research has been recognized as essential, to ensure the quality of translation and the cultural adaptation process [32–37], as well as to ensure equivalence between the original and the target language. This study aimed to promote a Brazilian cross-culturally adapted version of the English HCWM-RAT tool proposed by WHO [25]. It focused on equivalence of concepts, items and technical and semantic aspects, between the English and Brazilian Portuguese versions.

## Methods

### Instrument

The HCWM-RAT [25] is an assessment instrument consisting of a questionnaire with 85 items distributed over eight sections (toolboxes) containing 14 analysis criteria. It is structured as an electronic spreadsheet and has 8 supplementary sections: introduction, preparation, planning, contacts, glossary and abbreviations, personal observations, rating at the national level, and an inventory of all the questions.

The instrument contains five different options for answers: 1) multiple choice (C), which allows more than one option; 2) text (T),which allows open-ended answers; 3) numerical (N), which refers to the amount of generated waste and the size of the budget allotted to HCWM; 4) qualitative (Q), which allows ranking from 0 (nonexistent) to 5 (excellent); and 5) Boolean [B]: yes/no answers. The tool also provides space for the interviewer’s personal observations, to facilitate information matching. HCWM-RAT follows a logical and chronological frame, and can cover areas from the national level (ministries) to the local level (individual healthcare facilities) and considers all stakeholders involved in the issue of waste management [25].

### Cross-cultural adaptation

The universal approach of Herdman, Fox-Rushby and Badia [35], was chosen because that method “emphasizes the possibility of cross-cultural variations in the nature of multidimensional concepts” [p.324]. In addition, the approach of Beaton, Guillemin and Bombardier [32] was applied to carry out the cross-cultural adaptation. To assess both conceptual equivalence and items using the cross-cultural adaptation method, a thorough literature review was carried out. A theoretical reference was built and, subsequently, constructs related to an integrated system of HCWM were analyzed [6, 8, 10, 11, 38, 39]: “sustainable development”, “safe management”, “health-care waste”, “safe handling”, “environmental health”; and political, legal, technical and operational concepts in both cultures [4, 14, 16, 17, 22, 24, 40]. Moreover, the terms adopted by the major Brazilian databases of HCWM, such as the National Register of Health Care Facilities (CNEN) [21], the National Information System on Sanitation (SNIS) [41] and the Brazilian Institute of Geography and Statistics (IBGE) [42] were verified.

As suggested in the literature, the evaluation of conceptual equivalence and items was complemented by structural analysis of the original instrument, to determine whether its various dimensions would be relevant in the Brazilian context [32, 35]. The participation of a committee of experts and a target population of the study were used to improve this process [32, 33, 36]. The committee evaluated the semantic equivalence, which involved the formal analysis of all stages of the cross-cultural adaptation process [32, 33, 36].

**Fig. 1:**
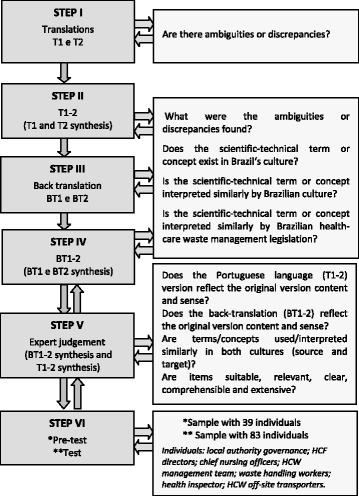
Steps of cross-cultural adaptation and guideline questions

Conceptual questions, shown in Fig. 1, were formulated to guide the study and to help the analysis, through the following steps:

- Step 1 (*Translation*): Two independent translations of the HCWM–RAT to Brazilian Portuguese (T1 and T2) were made by different certified bilingual professionals, named as Translator A and Translator B: one with previous knowledge of the theme and the other without it. This step was aimed at strengthening the possibility of finding badly formulated questions or linguistic ambiguities [32, 34–36].
- Step 2 (*Translation synthesis*): Discussions of translation differences were conducted during a meeting between the translators and the author of this study, using the Nominal Group Technique (NGT) [43] to produce a synthesis of the two translations (T1-2). This process of comparison was carried out taking into consideration the original instrument, the theoretical framework, and the political and regulational context in both languages and cultures. Because the original instrument contained a large number of sections and had a complex structure, a source spreadsheet incorporating translations T1 and T2 was created, to compare the translations, in order to produce a synthesis of the two. In this source spreadsheet, the lines were identified by different background colors, identifying the two translations, while the consensual synthesis was identified by a third color pattern. The main issues and the method for achieving consensus were described in reports produced by the author of this study, using methods recommended in the literature [32].
- Step 3 (*Back translation*): The back translation was performed to generate another version of the questionnaire (T1-2) in its original language (English). This process was conducted totally blind of the original version by two other different translators, whose mother tongue was English, named here Translator C and Translator D (native English-speaking back-translator, with a Doctorate in Public Health concluded in Brazil) as indicated in the methodology [32].
- Step 4 (*Back translation synthesis*): The two back translations (BT1 and BT2) were systematized into a consensual version during a meeting following the same methodology explained previously, producing a synthesis (BT1-2).
- Step 5 (*Expert judgment*): The semantic equivalence (connotative and denotative) [35, 36] was rated by a committee of experts, based on the three versions — the original (HCWM-RAT), T1-2, and BT1-2 — along with respective reports produced in the translation and synthesis steps [32].
  Representatives of different areas of healthcare waste management made up the committee of experts: researchers, and professionals in the fields of biosafety, hospital quality and management, occupational health, and the physics and hygiene of radiation. Translator D also took part in this group, since he was the only native speaker specialized in public health with a Doctorate in Brazil. There were also members of municipal hospitals, cleaning and hygiene staff, representatives of a municipal garbage collecting company, statistical experts, and representatives of the Sanitary Surveillance Agency, the General Coordination for Environmental Surveillance (Health Ministry) and the Brazilian Environmental Council (Environmental Ministry).
  A 2-day meeting was conducted, and since the HCWM system to be used is regulated by the government, participation from all aspects of HCW had to be obtained, to contribute positively and productively. The meeting employed a nominal group technique with audio recording [43]. The group evaluated the clarity, coherence and pertinence of all the items and sections in the tool, with respect to the Brazilian context. After analyzing the material and coming to consensus, the committee of experts, along with the author of this study, created the preliminary version to be submitted for a pre-test.
- Step 6 (*pre*-*test*): The pre-test was presented to 39 individuals [32], who were selected for their positions as representatives of government or of one of the nine public health care facilities of the Municipality of Niteroi, Rio de Janeiro; at least one individual was selected from each of the nine facilities. An individual could be included in the sample if he or she: performed a function in a governmental sector involved with health or the environment; was a health-care facility director or manager, or a nursing supervisor; was an HCWM commissioner; or was a worker involved with health-care waste handling.

The pre-test was conducted with a heterogeneous group of targeted interviewees in 3 different meetings (March 2011) of 3 h each; the interviewee had to analyze as well as answer the items. A single interviewer conducted this pretest.

To answer the *preliminary version* of the translated instrument, the individuals had to complete a form with two questions, to evaluate the quality of the items: 1) Is it easily understood? (Y/N); 2) Is it appropriate for collecting information on an HCWM system? (Y/N). For both questions, the interviewee was also asked to rate the comprehensibility of the item and to suggest changes for improvement. When the number of “no” answers to either question for a given item exceeded 5 %, that item was included in a list for further analysis and discussion among the experts. Because a number of confusing, ambiguous or inconsistent items were identified by the sample group, a second test was conducted 2 weeks later, by a single interviewer, asking the same questions of the revised item. This second test (re-test) occurred with 83 participants [32], including HCF professionals, government representatives, and others directly or indirectly involved in HCWM systems.

## Results

After analysis of the literature and regulations, and further discussions among the experts, the constructs in the original instrument were considered relevant and applicable to the Brazilian context. The concepts and dimensions were consistent with Brazilian HCWM policies, and included occupational health and safety, biosafety, ecology, and sanitation [16, 17, 24]. The 14 criteria of the original instrument were identified in the Brazilian Version of the HCWM-RAT and were grouped into five dimensions (Table 1).

**Table 1:** Dimensions and criteria of the health-care waste management – rapid assessment tool (HCWM-RAT) Brazilian version

Dimension	Criteria
Spatial and characterization of HCF	1 Geographic and demographic situations
	2 Health-carefacilities (HCF)
Capacity building, safety and health	3 Staff/Health professionals
Handling steps	4 Generation
	5 Segregation
	6 Internal container storage
	7 Internal storage area
	8 Internal collection and transportation
	9 Transportation to external locations
	10 Treatment
	11 Final disposal
Public policy and budgets	12 Regulations and guidelines
	13 Policies and budgets
Sanitation and waste water	14 Sanitation and waste water

After adjustment, the original instrument could be considered applicable to Least Developed Countries (LDCs) [13] and those without regulations or policies on HCWM. Both the committee and the target population recommended the cross-cultural adaptation as being useful in Brazil. Such a cross-cultural adaptation of HCWM-RAT [25] was new and unknown in Brazil.

The aim of the committee of experts was to make the instrument suitable for use in a monitoring and evaluation program in Brazil. Changes and adequacies have followed semantic equivalence to concepts, terms and expressions from the original WHO instrument [32, 35, 36].

After evaluation of the semantic equivalence, two different translations (T1 and T2) were obtained. There were no discrepancies between the two back-translations (BT1 and BT2). During the synthesis elaboration of the translated and back-translated versions (T1-2 and BT1-2), terms such as *scope*, *feedback*, *checklist*, *stakeholders* were maintained because of their current use in Brazil, in the same cultural context.

The introduction (basic assumptions and objectives) to the Brazilian instrument included a guidance statement from, and the electronic addresses, of the Brazilian regulatory authorities (Table 2). Table 3 shows the acronyms and terms used in three official databases: the Brazilian Institute of Geography and Statistics [42]; the National Register of Health Care Facilities [21]; the National Sanitation Information System [41].

**Table 2:** Evaluation of conceptual, technical and semantic aspects between the Brazilian Portuguese version of the health-care waste management tool - rapid assessment tool (HCWM-RAT) and its original English version

Original version	Final Brazilian version	Comments
SectionIntroduction		
*[…] improper management of wastes generated in health care facilities causes direct health impacts on the community, the personnel working in health care facilities, and on the environment. In addition, pollution due to inadequate treatment of waste can cause indirect health effects to the community.*	[…] o gerenciamento inadequado dos resíduos gerados pelos Estabelecimentos de Saúde (ES) pode provocar impactos diretos na saúde da coletividade, dos profissionais que trabalham nos ES e no ambiente. Ademais, a poluição oriunda do tratamento inadequado dos resíduos pode causar efeitos indiretos na saúde da coletividade.	The translation “na saúde da coletividade” (on the community), instead of the literal word, “da comunidade” was used because in Brazil the term “community” has the sense of a limited geographical area, inhabited by an indigent population (often referred to as a “favela” or a slum).
*An example of such a plan can be found at the following address:* www.healthcarewaste.org	Para auxiliar a implantação do Plano de Gerenciamento de Resíduos de Serviços de Saúde (PGRSS), foi elaborado o Manual de Gerenciamento de Resíduos de Serviços de Saúde, lançado pela Anvisa, que igualmente disponibiliza outras orientações relevantes que podem ser encontradas na página do Órgão: www.anvisa.gov.br.	Here, it is stated that the Brazilian Health Surveillance Agency (Anvisa) has developed a handbook to help implement the Health-care Waste Management Plan (HCWMP). The professional is invited to access the electronic page of the Agency, where other guidelines are also offered.
SectionGlossary		
*Incineration - The controlled burning of solid, liquid or gaseous wastes to produce gases and residues containing little or no combustible material.*	Incineração - Queima controlada de materiais em temperaturas acima de 800 °C, na presença de oxigênio, sendo os produtos finais desta queima, quando completa: dióxido de carbono, vapor d'água e cinzas	As suggested by the experts, the definition given by current Brazilian regulations was maintained: As stated by the Ministry of Environment, incineration is any process that applies temperatures above 800 °C, in the presence of oxygen, generating carbon dioxide, water vapor and ashes. A consensus was attained that this is not derived from the meaning given by the original version.
*Pharmaceutical waste - Consisting of/or containing pharmaceuticals. [Includes: pharmaceuticals expired, no longer needed; their containers, items contaminated by or containing pharmaceuticals (bottles, boxes…)].*	Resíduo químico-farmacêutico - Consiste de/ou contém produtos farmacêuticos, incluindo medicamentos (com validade vencida ou não mais necessários) e seus recipientes quando contaminados. Regulamentados como uma subcategoria do Grupo B (Resolução RDC ANVISA n° 306/04 e CONAMA n° 358/05) são aqueles caracterizados nos itens 11.11 e 11.12 da RDC Anvisa n° 306/04	As suggested by the target professionals (from the field test) and the experts, the terminology given by the Brazilian regulations and requirements was adopted. in Brazil, pharmaceutical wastes constitute a sub-category in the Group B (Chemical Residues) (Anvisa 306/04 and Conama358/05). Therefore, besides describing the sub-category, the item indicates regulations and their requirements for identifying medicines that are subject to special control. It is understood that this does not derive from the meaning (waste and classification) given by the original version.
*Radioactive health-care waste - Consisting of/or containing radioactive substances. [Includes: unused liquids from radiotherapy or laboratory research; contaminated glassware, packages or absorbent paper; urine and excreta from patients treated or pre-tested with unsealed radionuclides; sealed sources].*	Rejeito radioativo - materiais resultantes de atividades humanas que contenham radionuclídeos (não selados) em quantidades superiores aos limites de isenção especificados nas normas da Comissão Nacional de Energia Nuclear (CNEN) e para os quais a reutilização é imprópria ou não prevista.	The translation that expresses the concept accepted and adopted by the Brazilian National Nuclear Energy Commission (Cnen) was maintained. The term “waste” is translated as a “Rejeito” (reject) (something that cannot be reused or recycled), instead of waste (something that can be reused, recycled or transformed - for energy). The radioactive reject is described as resulting from activities that use any kind of radionuclide (unsealed) above the amounts considered as improper for reuse by CNEN.
*Recycling - A term embracing the recovery and reuse of scrap or waste material for manufacturing or other purposes.*	Reciclagem - Processo de transformação dos resíduos sólidos que envolve a alteração de suas propriedades físicas, físico-químicas ou biológicas, com vistas à transformação em insumos ou novos produtos.	The definition given by the current regulations (as mentioned above) was maintained. Recycling was described as a biological, chemical or physical transformation process, resulting in new products or raw materials (“cradle to grave” or “cradle to cradle”). It is understood that this encompasses the term and meaning given by the original version.
*Risk - Probability that a hazard will cause harm and the severity of that harm.*	Risco à saúde - probabilidade da ocorrência de efeitos adversos à saúde, decorrentes da exposição humana a agentes físicos, químicos, de acidente (especialmente relacionados aos perfurocortantes) e biológicos	Risk is described as the probability of the occurrence of adverse effects to health, resulting from human exposure to physical, chemical or biological agents. In the case of health care wastes, sharp objects are particularly important.
*Safety (sharps) box - A puncture proof/liquid proof container designed to hold used sharps safely during disposal and destruction.*	Recipientes para acondicionamento de perfurocortantes - recipientes rígidos, resistentes à punctura, ruptura e vazamento, com tampa e devidamente identificados, atendendo aos parâmetros referenciados na norma NBR 13853:1997 da Associação Brasileira de Normas Técnicas (ABNT-NBR)	This item describes the characteristics of the safety boxes for sharps (perfurocortantes). It is warned that the box has to be resistant to perforation, disruption and leaks, and should have a cap and identification. It also has to display conformity to the Brazilian Standards (ABNT-NBR 13853:1997).
*Sharps - Sharps are a subcategory of infectious health care waste and include objects that are sharp and can cause injuries.[Includes: syringe needles, scalpels, infusion sets, knives, blades, broken glass].*	Perfurocortantes - Materiais perfurocortantes ou escarificantes, tais como: Lâminas de barbear, agulhas, escalpes, ampolas de vidro, brocas, limas endodônticas, pontas diamantadas, lâminas de bisturi, lancetas; tubos capilares; micropipetas; lâminas e lamínulas; espátulas; e todos os utensílios de vidro quebrados no laboratório (pipetas, tubos de coleta sanguínea e placas de Petri) e outros similares	In Brazil, sharps are categorized as Group E - Perfurocortantes (Anvisa n° 306/04 and Conama n° 358/05). In the regulations, sharps are described as materials or objects with sharp edges, sharp tips or rigid snags able to produce cuts or to perforate human skin. Sharps can be razor blades, needles, scalpels, shattered glassware, lancets, capillary tubes, micropipettes, microscope slides, cover slips, spatulas, or odontological instruments.
*Waste management - All the activities - administrative and operational - involved in the handling, treatment, conditioning, storage, transportation and disposal of waste.*	Gerenciamento de resíduos - Constitui-se em um conjunto de procedimentos de gestão, planejados e implementados a partir de bases científicas e técnicas, normativas e legais, com o objetivo de minimizar a produção de resíduos e proporcionar aos resíduos gerados, um encaminhamento seguro, de forma eficiente, visando à proteção dos trabalhadores, a preservação da saúde pública, dos recursos naturais e do meio ambiente	The current Brazilian regulatory definition was maintained. Waste management is described as a group of management procedures, scientifically supported, based on legal regulations and standards, aiming to reduce these byproducts and give them an adequate and safe fate. It is further explained that the aim of waste management is to protect workers and to preserve public health, natural resources and the environment.

**Table 3:** Inclusion of terms, acronyms and assessment requirements: conceptual, technical and semantic evaluation

Final brazilian version	Comments/description
Terms	
Resíduos similares ao RSU - Resíduos que se supõe não apresentam risco biológico, químico, de acidente com perfurocortantes ou físico (relativos aos rejeitos radioativos), à saúde ou ao meio ambiente, podendo ser equiparados aos resíduos domiciliares	According to the experts, the term “domestic waste” applied in the original version does not exist in the Brazilian regulation, but the closest applicable term is “similar to urban waste” (RSU), These residues do not present any significant risk to human health or the environment.
Célula especial de RSS - Consiste na disposição dos RSS em locais que observem os critérios mínimos estabelecidos pela Resolução do Conselho Nacional de Meio Ambiente, n°. 358/2005	Existing conditions for final HCW disposal in some regions of Brazil. A special cell for the disposal of HCW is a location for the destination of wastes, following the criteria established in the Conama n° 358/05 Act.
Armazenamento temporário (interno) - Consiste na guarda temporária dos recipientes contendo os resíduos já acondicionados, em local próximo aos pontos de geração, visando agilizar a coleta dentro do estabelecimento e otimizar o deslocamento entre os pontos geradores e o ponto destinado à apresentação para coleta externa	This term is given by the Brazilian regulations (Anvisa n° 306/04) and adopted requirements. Internal temporary storage is a site abutting the location of residue generation, where the residues are transitorily stored in labeled containers. This is a restricted area that aims to facilitate the collection and its transportation to the final destination.
Transporte interno - Consiste no traslado dos resíduos dos pontos de geração até local destinado ao armazenamento temporário ou armazenamento externo com a finalidade de apresentação para a coleta	This term is in accordance with the Brazilian regulations. Internal transportation is the collection of residues within the unit and its routing to internal temporary storage (see previous item).
Destinação final - Processo decisório no manejo de resíduos que inclui as etapas de tratamento e disposição final.	The term “destination” encompasses the decision making about the handling of HCW, its treatment and final destination, or recycling and reuse where applicable (in agreement with the specialists and target professionals – the HCW management staff).
Acronyms	
IBGE - Instituto Brasileiro de Geografia e Estatística; SNIC - Sistema Nacional de Informações das Cidades; CNES - Cadastro Nacional de Estabelecimentos de Saúde; CCIH - Comissão de Controle de Infecção Hospitalar; SESMT - Serviço Especializado em Engenharia de Segurança e Medicina do Trabalho;	IBGE, SNIS and CNES refer to Brazilian databases that must be consulted in order to answer certain items: Criterion 1 – Geographical and Demographic Situation and Criterion 2 – Health-Care Facilities (HCF). Both criteria concern the “location and characterization of HCF”. IBGE is the main demographic information provider, because it is responsible for the Brazilian Census, gathering social, economic and administrative data; SNIS gathers information on management of water services and solid residue; CNES is responsible for quantitative information on the HCF, respective locations/addresses, data on available infra-structure, type of health services provided, specialized health services, inpatient beds and health professionals; CCIH is composed of a team of graduated health professionals, formally designated for hospital infection control; SESMT is regulated by a Brazilian Standard for the prevention of occupational accidents and diseases. Depending on the size of the unit and the number of employees, the SESMT team must be composed of one labor-safety physician, one labor-safety nurse, one labor-safety nurse technician, one labor-safety engineer and one labor-safety technician.
Assessment Requirements legend (data qualitative [Q])	
Não possui conhecimento algum sobre os riscos oferecidos pelos RSS às pessoas que os manuseiam: Inexistente = 0. Sabe que os resíduos oferecem riscos, todavia não sabe esclacer como ou o porquê: Crítico (baixo) = 1. Possui conhecimento contido na opção 1 e sabe que precisa, por exemplo, usar EPI/EPC, mas não o porquê e/ou se este é adequado: Insuficiente = 2. Possui conhecimento contido na opção 2 e compreende a adequação e importância de práticas de prevenção de riscos: Satisfatório = 3. Possui conhecimento contido na opção 3 e sabe como reagir frente à situação de risco ou acidente (tipo reativo): Bom = 4. Possui conhecimento contido na opção 4, mas também sabe como prevenir e é capaz de orientar quanto às práticas de manejo frente aos riscos oferecidos pelos RSS (tipo pró-ativo): Excelente = 5	No criteria have been attributed for [Q] in the original version – Item 304 (staff for HCW awareness: awareness of risks of person(s) handling HCW?). Instead of literal translation, the term “conhecimento” (awareness) was applied because regulations in Brazil enforce HCW generators to promote a continuous education program for the staff managing wastes. Thus, the following criteria have been created for the Brazilian version: “nonexistent = 0” – The staff has no knowledge about any of the risks presented by HCW; “critical (low) = 1” – The staff knows that waste presents risks, but cannot explain how or why; ‘Insufficient - 2’- The staff has the knowledge contained in option 1 and knows the need, for example, for wearing individual or collective protective equipment (EPI or EPC), but they do not know the reason why or whether is it adequate; “satisfactory - 3” - The staff has the knowledge contained in option 2 and understands the adequacy and importance of risk-prevention options; “good - 4” - The staff has the knowledge contained in option 3 and knows how to react when facing a risk/accident situation (reactive type); “excellent - 5” - The staff has the knowledge contained in option 4, and also cares about prevention and is capable of guiding others in practices for managing the risks presented by HCW (pro-active type)

Additional sections (e.g., glossary) provided the standardized terminology used by the health system and the Brazilian regulatory agencies. For example, terms related to health-care-waste handling were included according to the Brazilian regulatory systems, e.g.,: “Temporary storage”; “Similar to the solid urban waste” (RSU in Brazilian Portuguese); “Internal transportation” (Table 2).

Terms and items that made no sense in the Brazilian context or culture were replaced by ones commonly applied in the national information system database for health (CNES). For example, the item referring to healthcare facilities (HCF) “category” had to be changed to either “type of establishment” or “level of hierarchy” (Item 200, shown in Table 4) in accordance with HCF registration nomenclature in the Brazilian health-system database.

**Table 4:** Evaluation of item equivalence between the Brazilian HCWM-RAT version and the original HCWM-RAT version

Original	Final brazilian version	Comments
*200 - health care facility (HCF): which category is it (are they)?* [1] *ambulant service;* [2] *(sub-)district hospital*	Estabelecimento de saúde (ES): Qual é o tipo do estabelecimento e a que nível de hierarquia pertence*?* 1] ES para atenção básica; [2] ES de média complexidade; [3] ES de alta complexidade	The terms “type” (tipo) and “level hierarchy” (“nível de hierarquia”) are used in the Brazilian database (CNES) to register HCF to operationalize the Informational Systems in Health. The HCF “type” is defined based on the professional activities and the services offered to a population. The “hierarchy level” indicates the degree of complexity of services provided (basic health assistance, medium and high complexity).
201 - *HCF which type is it (are they)?* [1] *public;* [2] *private*	ES – De que natureza é? [1] público; [2] privado; [3] outros (especificar)	In the Brazilian database (CNES) the term “nature” (“natureza”) defines the origin of the organization share capital and the HCF administrative link. Option 3 “others (specify)” makes possible the identification of the different administrative conditions found in Brazil.
401 - *domestic waste: quantity produced/day (estimated, in kg or litres)*	resíduos similares aos sólidos urbanos (RSU): quantidade gerada por dia (estimativa, em kg ou litros)	According to experts the term “similar to urban waste” (RSU) is the closest term applicable, for Brazilian culture. This item refers to the quantity of waste generated per day that is capable of being recycled or reused.
404 - *anatomic waste: quantity produced/day (estimated, in kg or litres)*	peças anatômicas: quantidade gerada por dia/semana (estimativa, em kg ou litros)	The term “anatomic waste” was changed to “peças anatômicas” because in the target culture the term “waste” has a negative connotation (equivalent to “garbage”), which would be considered offensive when applied to human body organs or parts. The term “produced” denotes the interest in conceiving a product. The use of the word “gerada” gives the sense of a byproduct that results from any activity or procedure and that has no use. In the target culture HCW is generated, not produced.
405 - *pharmaceutical waste: quantity produced/day (estimated, in kg)*	Resíduo químico-farmacêutico: quantidade gerada por dia/semana (estimativa, em kg ou litros)	This item uses a terminology (“químico-farmacêutico”) suggested by the target population and the experts’ committee, to accommodate the difference in waste classification. Since “pharmaceutical” is not a Brazilian waste category, classifying such waste simply as category B, “chemicals” would include more than just pharmaceutical waste. This item can apply to the amount of pharmaceutical waste generated.
501 - *needle stick injuries: how many cases reported in the past 12 months*	ferimentos com perfurocortantes: quantos casos foram relatados nos últimos 12 meses?	This item uses the terminology used in Brazilian regulations (Anvisa n° 306/04 and Conama n° 358/05) to refer to Group E – “Perfurocortantes” waste. This item asks about the number of accidents that occurred during a year.
900 - *transport services: are there any control measures?* *[0] none;* [1] *transport form;* [2] *other (specify)*	serviços de transporte: há alguma medida de controle? [0] nenhuma; [1] forma de transporte; [2] emissão de documento de manifesto, CADRI,…; [3] outro (especificar)	In order to identify the measures of control used by the HCW transporters, two document examples were inserted: “manifesto” (waste transportation manifesto, used in the state of Rio de Janeiro) and “CADRI” (certificate of transportation of environmental interest waste, used in São Paulo state) as an option. These documents consist of legal ways to control and monitor the waste transported to treatment and disposal sites licensed by environmental organizations.
1007 - *domestic waste: how is it generally treated?*	resíduos similares aos RSU: geralmente, como são tratados (manejados - ogânico e recicláveis)?	In this item, a term in parenthesis, suggested by the experts’ committee and the target population during the field test, was included. In Brazil, the term “treated” is not applied to the category of domestic waste, which is sent to reprocessing. In order to preserve semantic equivalence, the term “treated” was changed to the “way of handling” “similar to urban waste” (RSU) (“manejo – orgânico e recicláveis”).
1202 - national HCWM regulations: does their application cause any problems ?	regulamentações nacionais para GRSS: a aplicação da regulamentação gera algum tipo de situação-problema?	Suggestion of the target population (during the field test) and the experts’ committee: In the Brazilian cultural context, the word “problem” relates to a negative condition obtained by following the regulation. Hence, the term was changed to “issue” (“situação-problema”). In the re-test this item was evaluated as capable of allowing conflicts to emerge during HCWM regulations application.

According to the committee of experts, all the items that could be answered through research in databases were listed in a separate data sheet to be collected prior to the interviewer’s field visits.

The dimension “Capacity building, safety and health” (Table 1) was included with some extra terms (Table 3, e.g., Item 304) to refer to the HCW handlers’ level of risk awareness; the original instrument did not offer parameters to classify this awareness level.

The dimension “Handling steps” was also changed to adapt to Brazilian culture. In Criteria 4 (generation) and 10 (treatment) the term “anatomical waste” was changed to “anatomical parts” (“peças anatômicas” in Brazilian Portuguese) because in the target culture the term “waste” has a negative connotation (equivalent to “garbage”), which would be considered offensive when related to human body organs or parts.

In addition, after a preliminary field version of the test, some items — for example, those referring to budget allocations for HCWM (found in Criterion 13 “Policy and budgets”) — were directed to multiple actors from different levels, a difference from the original version. The aim here was to better understand the difficulties that the HCWM staff and decision makers usually face when trying to interpret or verify rules, especially when they are not included in the decision-making processes. The suggestions and observations made by the people interviewed during the test phase were extremely helpful in producing the final version of the instrument.

## Discussion

Despite the conventions signed by Brazil, such as the Basel Convention [29], the Stockholm Convention [44] and the Minamata Convention [45], and their associated regulation structures and databases (CNES; SNIS; IBGE), the segregation and collecting of health-care wastes are still primitive in most Brazilian cities.

This lack of development contributes to the paucity of knowledge about the total amount of waste generated in health-care facilities, and its real destination, in Brazil [46].

Some studies have identified the importance of training programs directed to health workers, HCW management teams and waste handling workers in order to improve the global approach on HCWM [47, 48]. However, this has been neglected in Brazil [23] and may be one of the reasons behind the difficulties on HCWM faced by this country.

The use of periodic assessments supported by a comprehensive instrument that is adapted to Brazilian context and validated in the target country (Brazil), helps not only to identify problems but also to explain its causes providing decision makers with the necessary evidence to reorient strategies.

The cross-cultural adaptation process is an approach that can be applied to many instruments developed in other cultural and linguistic settings. For Brazil, it may help to fill the data gap about the critical knots for HCWM improvement as well as to provide feedback on HCW to DATASUS database.

The objective of the adaptation is to achieve equivalence between the original measurement instrument and its adapted version [32, 35]. Therefore, the Brazilian Portuguese version of the HCWM-RAT was obtained through careful cross-cultural adaptation steps, recommended in the literature. In contrast with other cross-cultural adaptation studies that focused on epidemiological measurement instruments, this research considers an equivalence study on an environmental health measurement instrument that, as applied, will help identify and make comprehensible the HCWM framework in Brazil.

In this cross-cultural research, the constitution of a committee of experts was shown to be fundamental for the achievement of equivalence, as well as the validity of the construct, the content and the face (apparent) of the adapted instrument [32, 34, 35, 37].

While it is recognized that additional tests for evaluating the instrument’s psychometric properties are highly recommended, they are not compulsory for the validation of the translated version [32]. However, to reinforce the process of version evaluation, a 16-member committee of experts was put together, composed of researchers, one of the translators, and representatives from one of the Brazilian HCWM regulation agencies. Using the NGT [43] with the support of a moderator, this evaluation was aimed at maximizing information compilation and encouraging experts to express their opinions, while avoiding any particular expert’s domination in the discussion.

Throughout the experts’ meeting, items were examined with the goal of reaching a consensus on each item before moving on to the following one. During this process, a theoretical construct coherent with universal principles [4] yet specific to the HCWM status investigation practice [4, 16, 17] was observed.

For both the conceptual and the item-level equivalence, a consensus of keeping the original structure and items prevailed, based on the instrument *modus operandi*: that is, it must be used only by trained interviewers — HCWM and M&E professionals. This recommendation was also stated in the introduction to the original instrument. For instance, the maintenance of item 1400 in the Brazilian version (“Do all patients have access to/use of toilets in the healthcare facility?”) drew a great deal of attention during the expert discussion, motivating 3 rounds using the NGT [43]. Ultimately, however, the experts decided that this item should be kept as it is, and as the Brazilian instrument is applied, the interviewers would evaluate the condition of the toilets provided by each facility. This decision was attained after the group agreed that Brazil is a huge country with 283,434 health-care facilities [21], in many different socio economic and cultural scenarios, and that therefore this item would be useful for identifying infrastructure shortcomings.

The preliminary version that was obtained after 2 days of expert meetings was tested with 39 individuals [32] from the target population, with the aim of adjusting the instrument to achieve equivalence between the original source and the target version (Brazilian) in different aspects involving clarity, coherence and pertinence of the questions. The test showed that some items still received more than 5 % of negative answers for quality evaluation (again using Step 6 of the method). In other words, although the preliminary version of the Brazilian HCWM-RAT had been thoroughly evaluated by experts, eight (8) individuals of the target population considered that some items were not clear, coherent or pertinent. Consequently, some questions were modified. For example, the item referring to “national HCWM regulations,” with the question “Does their application cause any problems?” was changed to “Does the application of the established rules generate any issues?" (“a aplicação da regulamentação gera algum tipo de situação-problema?” in Brazilian Portuguese): Item 1202 in the Brazilian Portuguese version, shown in Table 4). The experts claimed that the word “problem” could not be applied to the Brazilian context since it has a negative connotation and a regulation is not designed to cause negative effects but to help guide the population.

Another example raised by the target population was in Criterion 10, where it is asked how “urban solid waste” (in place of “domestic waste,” in the original instrument) is usually treated in a health-care facility. For this population the term “treated” was not coherent because it implied the need for a method, technique or process to reduce or eliminate any inherent contamination risk, occupational accident, or environmental damage. Therefore, in order to avoid semantic discrepancies between the original and translated versions, this item was changed to “how the waste is handled - organic and recyclable” (“como são manejados – orgânicos e recicláveis” in Brazilian Portuguese), as shown in Table 4.

After the modifications applied from the testing of the preliminary version, all items received a positive evaluation, indicating that interviewees had no difficulties in understanding the questions. Table 4 shows the original items and the translated and adapted ones, in the field tests and from the committee of experts’ analysis.

Reports of the revisions were written and sent to the WHO, explaining the rationale behind the decisions that resulted in changes in the adapted version: a necessary step for the official recognition of the Brazilian version of HCWM-RAT.

Operational equivalence was evaluated during both the test and the retest; but the method of administration and the estimated time for application of the tool remained the same as for the original instrument. With emphasis on the *modus operandi*, equivalence refers to a comparison between the characteristics of an instrument for use in target populations (Brazilian version) and of one for use in the original population source (source instrument) [36].

The Brazilian version of HCWM-RAT has the potential to generate indicators and official database feedback, and to subsidize political decisions at different political levels among decision makers. However, an investigation of the psychometric properties of the instrument should be performed in the future [32, 33, 36].

Although a Brazilian version of HCWM-RAT has been approved, there is no guarantee that the cross-cultural adaptation was effective without the assessment of measurement equivalence, therefore, sophisticated statistical methods such as item response theory model (IRT) can be used to confirm the results of this study and it can be considered as a limitation of the study.

From this study arises the possibility for the application of the instrument to be expanded to other Portuguese-speaking countries, considering the results of Step 2 (*Translation synthesis*, *T1*-*2*) of the cross-cultural adaptation process.

For countries that already have guidelines and regulations on HCWM, this study may at least reduce the effort required for the research and development of their own adapted versions.

## Conclusion

The results of this study show that developing a Brazilian version of HCWM-RAT can open new research paths and possibilities for expanding the comprehension of the HCWM system, as well as the critical factors to achieve proper HCW management. These factors are considered essential for the success of any HCWM Plan.

It thus supports decision-making and stimulates innovation in evaluation, for this specific field.

From this study also raises the possibility for the application of the instrument (Translation synthesis, T1-2) to other Portuguese-speaking countries. For countries that already have framework directive on HCWM, this study may at least reduce the effort required for the research and development of their own adapted versions.

## Abbreviations

CNES: National register of health care facilities
HCF: Health-care facility
HCW: Health-care waste
HCWM: Health-care waste management
HCWMP: Health-care waste management plan
HCWM-RAT: Health-care waste management-rapid assessment tool
IBGE: Brazilian Institute of Geography and Statistics
NGT: Nominal group technique
REM: rapid evaluation method
SNIS: National information system on sanitation
SUS: Unified health system
WHO: World Health Organization

## References

[1] Forastiere F, Badaloni C, Hoogh K, Kraus MK, Martuzzi M, Mitis F, Palkovicova L, Porta D, Preiss P, Ranzi A (2011). Health impact assessment of waste management facilities in three European countries. Environ Health.

[2] Prüss-Üstün A, Rapiti E, Hutin Y (2005). Estimation of the global burden of disease attributable to contaminated sharps injuries among health-care workers. Am J Ind Med.

[3] Saia M, Hofmann F, Sharman J, Abiteboul D, Campins M, Burkowitz J, Choe Y, Kavanagh S (2010). Needlestick injuries: incidence and cost in the United States, United Kingdom, Germany, France, Italy, and Spain. Biomedicine International.

[4] Chartier Y, Emmanuel J, Pieper U, Prüss A, Rushbrook P, Stringer R, Townend W, Wilburn S, Zghondi R (2014). Safe management of wastes from health-care activities.

[5] Nascimento TC, Januzzi WA, Leonel M, Silva VL, Diniz CG (2009). Occurrence of clinically relevant bacteria in health service waste in a Brazilian sanitary landfill and antimicrobial susceptibility profile. Rev Soc Bras Med Trop.

[6] Fuerhacker M (2009). EU Water Framework Directive and Stockholm Convention: can we reach the targets for priority substances and persistent organic pollutants?. Environ Sci Pollut Res Int.

[7] Koloutsou-Vakakis S, Chinta I (2011). Multilateral environmental agreements for wastes and chemicals: 40 years of global negotiations. Environ Sci Tech.

[8] Singh S, Prakash V (2007). Toxic Environmental Releases from Medical Waste Incineration: A Review. Eviron Monitor Assess.

[9] Porta D, Milani S, Lazzarino AI, Perucci CA, Forastiere F (2009). Systematic review of epidemiological studies on health effects associated with management of solid waste. Environ Health.

[10] Hossain MS, Santhanam A, Nik Norulaini NA, Omar AK (2011). Clinical solid waste management practices and its impact on human health and environment – A review. Waste Manag.

[11] Harhay MO, Halpern SD, Harhay JS, Olliaro PL (2009). Health care waste management: a neglected and growing public health problem worldwide. Trop Med Int Health.

[12] Georgescu C (2011). Report of the Special Rapporteur on the adverse effects of the movement and dumping of toxic and dangerous products and wastes on the enjoyment of human rights.

[13] UNCTAD. World Investment Report 2013. Global Value Chains: Investment and Trade for Development. United Nations Conference on Trade and Development. 2013.

[14] Prüss-Üstün A, Giroult E, Rushbrook P (1999). Safe management of wastes from health-care activities.

[15] Karliner J, Guenther R (2011). Global green and healthy hospitals: a comprehensive environmental health agenda for hospitals and health systems around the worl.

[16] Ministry of Health, Brazilian Health Surveillance Agency. Regulation about health-care waste management. In: Brazilian Health Surveillance Agency, Regulation no. 306/2004, Dec 7th, editor. 306/2004. 2004th ed. Brasília: Ministry of Health; 2004.

[17] Ministry of the Environmental, National Environment Council., National Environment Council, Regulation no. 358/2005, Apr 29th (2005). Regulation about health-care waste treatment and disposal. 358/2005.

[18] Ministry of Science Technology and Innovations. Brazilian Nuclear Energy Commission. Radioactive waste management in radioactive facilities. In: Brazilian Nuclear Energy Commission, Norm CNEN-NE.6.05/1985. Brasília: Ministry of Science Technology and Innovations; 1985.

[19] DATASUS. Health information database from SUS. Brasília: Ministry of Health. http://datasus.saude.gov.br/informacoes-de-saude. Accessed 1 Sept 2015.

[20] Becerril-Montekio V, Medina G, Aquino R (2011). Sistema de salud de Brasil. Salud Publica Mex.

[21] Secretaria de Atenção à Saúde. Cadastro Nacional de Estabelecimentos de Saúde. Brasília: Ministry of Health; 2014. http://cnes2.datasus.gov.br/Mod_Ind_Unidade.asp. Accssed 1 Sept 2015.

[22] Brazil. Política Nacional de Resíduos Sólidos. In: Environment Mot, 12.305/2010, editor. 12305. Brasília: Minitry of the Environment; 2010. http://www.planalto.gov.br/ccivil_03/_ato2007-2010/2010/lei/l12305.htm. Accessed 1 Sept 2015.

[23] Moreira AMM, Günther WMR (2013). Assessment of medical waste management at a primary health-care center in São Paulo, Brazil. Waste Manag.

[24] Brazilian Health Surveillance Agency, Ministry of Health. Manual de gerenciamento de resíduos de serviços de saúde, Série A - Normas e Manuais Técnicos. Brasília: Ministry of Health; 2006. http://www.anvisa.gov.br/servicosaude/manuais/manual_gerenciamento_residuos.pdf. Accessed 1 Sept 2015.

[25] Health-care waste management - Rapid assessment tool for country level. [http://www.who.int/water_sanitation_health/medicalwaste/ratupd05.pdf]. Accessed 1 Sept 2015.

[26] Mcnall M, Foster-Fishman PG (2007). Methods of Rapid Evaluation, Assessment, and Appraisal. Am J Eval.

[27] Anker M, Guidotti RJ, Orzeszyna S, Sapire SA, Thuriaux MC. Rapid evaluation methods (REM) of health services performance: methodological observations. Bull World Health Organ. 1993;71(1):15–21. PMC2393426 8440033

[28] Rossi PH, Freeman H, Lipsey MW (1999). Evaluation – a systematic approach.

[29] UNEP. Basel convention on the control of transboundary movements of hazardous wastes and their disposal. United Nations Environmental Program. 2014. Accessed 1 Sept 2015.

[30] Stelzig S. Country Profile: Brazil. Focus Migration. 2008;15:1–11.

[31] IOM. Migration Initiatives 2015: Regional Strategies. Migrants and Cities. International Organization for Migration. 2015.

[32] Recommendations for the crosscultural adaptation of the DASH & quickDASH outcome measures. [http://www.dash.iwh.on.ca/assets/images/pdfs/X-CulturalAdaptation-2007.pdf]. Accessed 1 Sept 2015.

[33] Gjersing L, Caplehorn JRM, Clausen T (2010). Cross-cultural adaptation of research instruments: language, setting, time and statistical considerations. BMC Med Res Methodol.

[34] Guillemin F (1995). Cross-cultural adaptation and validation of health status measures. Scandinavian Journal of Rheumatolology.

[35] Herdman M, Fox-Rushby J, Badia X (1998). A model of equivalence in the cultural adaptation of HRQoL instruments: the universalist approach. Qual Life Res.

[36] Reichenheim ME, Moraes CL (2007). Operationalizing the cross-cultural adaptation of epidemological measurement instruments. Rev Saude Publica.

[37] Sperber AD (2004). Translation and validation of study instruments for cross-cultural research. Gastroenterology.

[38] Silva ENC, Costa MAF, Costa MFB (2009). Resíduos de Serviços de Saúde: aspectos conceituais, legais e técnico- operacionais. Biossegurança geral para cursos técnicos da área de saúde.

[39] Morelli J (2011). Environmental Sustainability: A Definition for Environmental Professionals. Journal of Environmental Sustainability.

[40] IPEN. International POPs Elimination Network. International POPs Elimination Project. Mobilizing Brazilian Civil Society for Stockholm Convention Implementation. Brazil: International POPs Elimination Network. 2006. Accessed 1 Sept 2015.

[41] Sistema Nacional de Informações sobre Saneamento. [http://www.snis.gov.br]. Accessed 1 Sept 2015.

[42] Demographic census 2010. [http://ces.ibge.gov.br/base-de-dados/metadados/ibge]. Accessed 1 Sept 2015.

[43] Totikidis V (2010). Applying the nominal group technique (NGT) in community based action research for health promotion and disease prevention. The Australian Community Psychologist.

[44] United Nations Environmental Program - UNEP, ONU (2001). Stockholm Convention on Persistent Organic Pollutants. Promulgated by Federal decree n° 5.472, on June 20th, 2005.

[45] UNEP. Minamata Convention on Mercury. United Nations Environmental Program. 2013. Accessed 1 Sep 2015.

[46] ABRELPE. Panorama dos Resíduos Sólidos no Brasil. São Paulo: Associação Brasileira de Empresas de Limpeza Pública e Resíduos Especiais -; 2013. http://www.abrelpe.org.br/panorama_edicoes.cfm. Accessed 1 Sept 2015.

[47] Kumar R, Somrongthong R, Shaikh BT (2015). Effectiveness of intensive healthcare waste management training model among health professionals at teaching hospitals of Pakistan: a quasi-experimental study. BMC Health Serv Res.

[48] Thakur V, Ramesh A (2015). Healthcare waste management research: A structured analysis and review (2005–2014). Waste Manag Res.

